# Simple tools for projecting patient recruitment

**DOI:** 10.1186/1745-6215-14-S1-P137

**Published:** 2013-11-29

**Authors:** Paul Silcocks

**Affiliations:** 1 CRUK Liverpool Cancer Trials Unit, Liverpool, UK

## 

Poor recruitment to clinical trials is a well-recognised and serious problem. Various predictors of poor recruitment have been identified and of course should be accounted for in trial design. Trial planning however will need a realistic projection of sample recruitment that also allows for random variation – yet available information may be minimal early in the development of a proposal. Such projections are also required when estimating power for survival analyses (the yield of events depends both on numbers and timing of patient accrual) – for example if using the Stata add-on artsurv.

I show here how minimal information, simple formulae and some reasonable assumptions can be combined to estimate both a sensible projection of expected numbers and a plausible range of variation; in turn this variation indicates the extent to which achieving the target sample size may be delayed. Simple calculations also indicate potential gains from restricting eligibility to better-performing sites and the relative lack of benefit from opening better-performing sites first.

